# Electroporation outperforms in vivo-jetPEI for intratumoral DNA-based reporter gene transfer

**DOI:** 10.1038/s41598-020-75206-2

**Published:** 2020-11-11

**Authors:** Liesl Jacobs, Elien De Smidt, Nick Geukens, Paul Declerck, Kevin Hollevoet

**Affiliations:** 1 Laboratory for Therapeutic and Diagnostic Antibodies, KU Leuven – University of Leuven, Campus Gasthuisberg O&N II, Herestraat 49 Box 820, 3000 Leuven, Belgium; 2 PharmAbs – the KU Leuven Antibody Center, KU Leuven – University of Leuven, Leuven, Belgium

**Keywords:** Cancer therapy, Reporter genes, Bioluminescence imaging, Gene therapy, Nucleic-acid therapeutics

## Abstract

Intratumoral delivery of drug-encoding plasmid DNA (pDNA) enables localised in vivo expression of biological drugs, offering an attractive alternative to conventional protein treatment. However, this requires physical or chemical methods to enhance the low transfection efficiency of naked pDNA. Electroporation and complexation with the polycation in vivo-jetPEI are both evaluated in the clinic for intratumoral pDNA delivery, but lack head-to-head comparison. This study therefore compared both methods for intratumoral DNA-based reporter gene transfer in a subcutaneous mouse tumour model. Intratumoral electroporation resulted in strong reporter expression that was restricted to the tumour area and persisted for at least ten days. Intratumoral expression after injection of pDNA-jetPEI complexes was two to three logs lower, did not exceed the background in most mice, and lasted less than five days even with repeated dosing. Remarkably, reporter expression was primarily detected in the lungs, presumably due to leakage of pDNA-jetPEI complexes into the systemic circulation. In conclusion, electroporation enabled more efficient, prolonged and tumour-specific reporter expression compared to intratumoral injection of pDNA complexed with in vivo-jetPEI. These results favour the use of electroporation for intratumoral DNA-based gene transfer, and suggest further optimisation of pDNA-jetPEI complexes is needed to improve their efficacy and biosafety.

## Introduction

Gene transfer of biological drugs presents an attractive alternative to conventional treatment modalities. Delivery of the drug-encoding nucleotides enables the patient’s body to express the drug in vivo for a prolonged period of time, bypassing the complex in vitro production and frequent parenteral administration of purified proteins^1^. Intratumoral gene transfer, for example, allows local anti-cancer therapy, thereby limiting systemic drug exposure and associated toxicity^2^. We recently demonstrated preclinical proof of concept for intratumoral delivery of DNA-encoded immunomodulatory antibodies^3^ and cancer-targeting nanobodies^4^. Current clinical applications include intratumoral DNA-based gene transfer of cytokines (e.g. NCT01502293), cancer vaccines (e.g. NCT04160065) and suicide genes (e.g. NCT00711997).

In addition to plasmid DNA (pDNA), other vectors have been used for intratumoral gene transfer^2^. While recent studies demonstrated promising results with mRNA, including fast but transient gene expression, most preclinical and clinical data have been reported with viral vectors. The latter enable robust and prolonged production of the transgene, with oncolytic viruses giving the additional advantage of tumour-specific cell killing and immune activation. However, pre-existing or induced immunity towards the viral vector often complicates their translation to the clinic^1,2^.

Compared to viral vectors, pDNA is much less immunogenic, is easier to produce and has no defined restrictions regarding the size of the transgene. pDNA therefore presents a convenient expression platform for biological drugs, yet its low transfection efficiency requires physical or chemical methods to enhance in vivo pDNA uptake^1,2^. Electroporation, for example, employs localised electrical pulses to generate transient pores in cell membranes^5^. Cationic carriers such as the commercially available polyethylenimine in vivo-jetPEI form complexes with pDNA, thereby protecting it against degradation and promoting cellular entry by endocytosis^6^. Both transfection methods have their advantages and disadvantages from a practical point of view. Compared to chemical methods, electroporation does not involve any specific pDNA formulation or preparation and is generally considered as simple and straightforward. However, it does require a dedicated medical device for electrical pulse delivery, which could give synthetic formulations a broader application potential.

Electroporation and in vivo-jetPEI are both evaluated in the clinic for intratumoral gene transfer^5–8^. However, to the best of our knowledge, preclinical and clinical head-to-head comparison of both methods is lacking for this application, despite comparative reports for other administration routes^9,10^. The current study therefore compares intratumoral electrotransfer of naked reporter pDNA with intratumoral injection of reporter pDNA complexed with in vivo-jetPEI in mice.

## Results

The efficiency of electroporation- and polymer-based transfection was evaluated by means of intratumoral reporter gene transfer in C57BL/6J mice bearing a subcutaneous MC38 tumour. A *firefly* luciferase (*f*luc)-encoding plasmid (pFluc) was delivered, which allowed both localisation and quantification of the resulting reporter expression in vivo and ex vivo. Electroporation pulse parameters were extracted from the literature^11^ and recently validated in-house^3,4^. The pDNA-jetPEI complexes were prepared and administered according to the instructions of the manufacturer (Polyplus-transfection).

**Figure 1:**
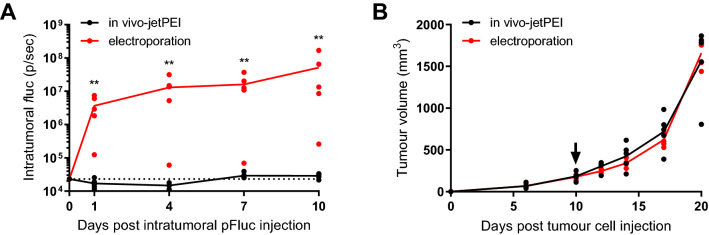
Comparison of electroporation and in vivo-jetPEI for intratumoral DNA-based reporter gene transfer. C57BL/6J mice received a single intratumoral electrotransfer of 20 µg naked pFluc or a single intratumoral injection of 20 µg pFluc complexed with 3.2 µl in vivo-jetPEI, ten days after MC38 tumour cell injection. (**A**) Intratumoral *f*luc expression. Data were compared between both groups at different time points with Mann–Whitney tests (n = 5 mice per group, **p < 0.01). The dots indicate the individual *f*luc expression in each mouse, the solid lines the mean expression in each group, and the dotted line the mean bioluminescence background in untreated mice. (**B**) MC38 tumour growth. According to Mann–Whitney tests, the difference in tumour volume between both groups was not significant at all time points (n = 5 mice per group). The dots indicate the individual tumour volume for each mouse, the solid lines the mean volume for each group, and the arrow the time of intratumoral pFluc injection. p/sec: photons per second.

Intratumoral electroporation of 20 µg pFluc led to strong reporter expression, which was restricted to the tumour area and did not decline for at least ten days, until the mice had to be sacrificed because of increased tumour volume. Intratumoral injection of the same pFluc dose complexed with in vivo-jetPEI gave *f*luc signals virtually similar to the background in untreated mice, and 200- to 1,800-fold lower compared to the signals after intratumoral electroporation (Fig. 1A, *p* = 0.0079). Both transfection methods had a similar effect on tumour growth (Fig. 1B).

**Figure 2:**
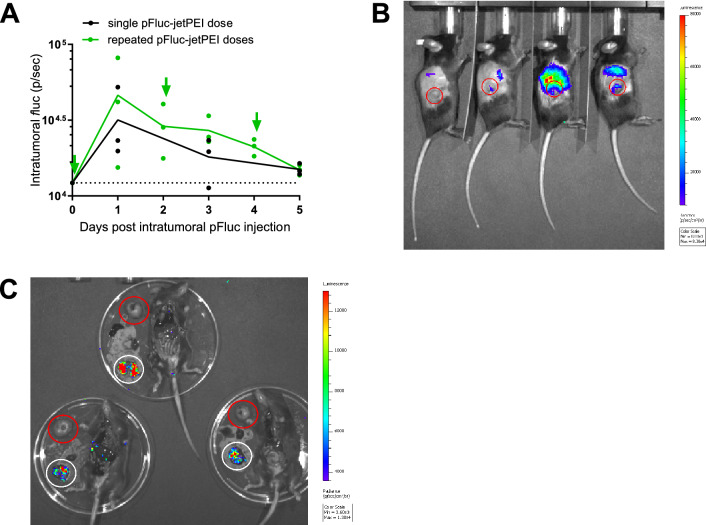
Single and repeated intratumoral administration of reporter pDNA complexed with in vivo-jetPEI. C57BL/6J mice received one or three intratumoral injections of pFluc-jetPEI complexes with the first dose ten days after MC38 tumour cell injection. (**A**) Intratumoral *f*luc expression over time. According to Mann–Whitney tests, the difference in expression between both groups was not significant at all time points (n = 3 mice per group). The dots indicate the individual *f*luc expression in each mouse, the solid lines the mean expression in each group, the dotted line the mean bioluminescence background in untreated mice, and the arrows the intratumoral pFluc-jetPEI injections. The injections on day 2 and 4 were performed shortly after bioluminescence imaging. (**B**) In vivo bioluminescence image of *f*luc expression in the repeated-dosing group, one day after the first pFluc-jetPEI injection. Next to the low *f*luc expression in the tumour area (indicated by the red circles), a clear signal was visible at the chest region. The third mouse showed aberrantly high off-target expression, interfering with the determination of the intratumoral *f*luc signal. This mouse is therefore not included in the graph in panel A, which is substantiated by a positive Grubbs’ test for outliers. (**C**) Ex vivo bioluminescence image of different organs of mice in the repeated-dosing group, ten days after the first pFluc-jetPEI injection. Red circles indicate the tumours, and white circles the lungs. The upper mouse, the lower mouse on the left and the lower mouse on the right correspond to the third, fourth and first mouse in panel B, respectively. p/sec: photons per second. p/sec/cm^2^/sr: photons per second that leave a square centimetre of tissue and radiate into a solid angle of one steradian.

To improve in vivo transfection, repeated intratumoral pFluc-jetPEI dosing was evaluated and compared to a single pFluc-jetPEI injection. In this experiment, pFluc-jetPEI did enable clear but low bioluminescence signals at the tumour in three out of six mice. However, the signal dropped after one day and reached background values by day five in both treatment groups (Fig. 2A). One mouse exhibited aberrantly high off-target expression that interfered with the measurement of the intratumoral *f*luc signal, and was therefore excluded in the analyses. Remarkably, also in other mice, the highest *f*luc expression was detected outside the tumour area at the chest region (Fig. 2B). This expression pattern suggested transfection of the lungs, which was confirmed by ex vivo imaging of different organs (Fig. 2C). We hypothesise that this was caused by leakage of pFluc-jetPEI in the systemic circulation, since it has previously been shown that intravenous injection of cationic systems like in vivo-jetPEI lead to high intrapulmonary expression^12^. With electroporation, naked pFluc can also leak into the bloodstream, but transfection is restricted to the area of the applied electrical field^3, 5, 13^, as further confirmed in the current study.

## Discussion

Intratumoral gene transfer has shown promising results for various biological drugs in both preclinical and clinical trials. Most results to date are based on the use of viral vectors^2^, despite recent progress with intratumoral pDNA delivery^3,7,8,14^. Comparison of some of the most used pDNA transfection methods may guide future research to further improve the efficacy of DNA-based intratumoral gene transfer and advance the non-viral field.

In this preclinical study, we report the comparison of two intratumoral pDNA transfection methods: electroporation and complexation with the polycation in vivo-jetPEI. Both methods were compared by means of bioluminescence imaging of *f*luc expression, which provides a straightforward and quantitative readout. Whereas the results with electroporation were in line with the literature^3,13^, pDNA-jetPEI complexes failed to efficiently transfect the tumour, even with repeated dosing. This is in contrast with the anti-tumour responses observed after jetPEI-driven intratumoral gene transfer in some preclinical and clinical trials^8,14^. Interestingly, Ohlfest et al*.* also reported very limited intratumoral reporter expression with pDNA-jetPEI complexes, but were able to improve transfection by reducing the speed of injection (from 100 µl in > 5 s to 100 µl in 60 s). However, delivery with a micropump at 10 µl/min again reduced pDNA transfection^15^. Coll et al*.*, on the other hand, showed that intratumoral reporter expression could be increased up to tenfold by switching from manual to micropump-assisted (20 µl/min) intratumoral injection of pDNA complexed with a linear polyethylenimine, and by adapting the N/P ratio (from 5 or 20 to 10), which represents the number of nitrogen residues of the polyethylenimine per pDNA phosphate group^16^. In the current study, only one setup was evaluated for the pDNA-jetPEI complexes (N/P ratio of 8 and injection of 50 µl in less than 20 s). Still, it is uncertain that even with additional improvements, in vivo-jetPEI could match electroporation, as the latter outperformed in vivo-jetPEI by a factor of 200 to 1,800 in our study.

A few studies describe the spatial distribution of reporter expression after intratumoral DNA-based gene transfer. Both electroporation and in vivo-jetPEI have been shown to enable expression restricted to the tumour site^13,15^. In the current study, however, clear transfection of the lungs was detected in multiple mice after intratumoral pDNA-jetPEI injection, whereas expression following electroporation was always limited to the tumour area. Even though based on a limited data set, these results suggest that considerable attention must be given to pDNA distribution when using pDNA-complexing agents. In addition to improving transfection, as suggested above, slower injection could increase the retention of the pDNA complexes in the tumour and reduce leakage into the circulation^16^. Modification of cationic carriers with e.g. tumour-targeting peptides or the use of tumour-specific promoters could further avoid off-target transgene expression, reducing the risk of systemic toxicity with polymer-based intratumoral gene therapy^12^.

The conclusions of this study need to be interpreted with caution as they are based on a restricted number of experiments in a single tumour model, with a single pDNA-jetPEI composition and a single gene expression readout. Nevertheless, despite the small sample sizes, clear and significant differences were observed between electroporation- and jetPEI-driven transfection, which were consistent across experiments. Future studies may be considered to evaluate if these conclusions can be extrapolated to a general context, e.g. by comparing both transfection methods in additional tumour models. In follow-up of the similar tumour growth observed with both transfection methods, a more thorough assessment of their impact on cell viability could be considered. For the pDNA-jetPEI complexes, the composition and way of administration may be further optimised. However, as mentioned earlier, it is uncertain that these improvements will be sufficient to match the performance of electroporation.

In summary, we demonstrated that electroporation resulted in stronger, longer-term and more localised expression compared to pDNA complexation with in vivo-jetPEI, when applied for intratumoral DNA-based reporter gene transfer. This confirms the potential of electroporation, and illustrates that further optimisation of the in vivo-jetPEI complexes is required to allow efficient tumour-specific pDNA transfection.

## Methods

### Plasmid DNA

pFluc was obtained from Icosagen (Tartu, Estonia). This pDNA construct encodes *firefly* luciferase 2 under control of a CAG promoter, and contains a backbone comprising an ampicillin resistance gene and pUC origin of replication. pFluc production and purification was performed as described previously^17^, except that pFluc was eluted with sterile milliQ water instead of D-PBS.

### Mouse tumour model

The MC38 murine colon cancer cell line was purchased from Kerafast (ENH204-FP, Boston, MA, USA) in March 2017 and was shown to be free of *Mycoplasma*. Cells were grown in Dulbecco's Modified Eagle Medium, supplemented with 10% heat-inactivated foetal bovine serum, 0.1 mM non-essential amino acids, 1 mM sodium pyruvate, 10 mM HEPES and 50 U/ml penicillin/streptomycin (Thermo Fischer Scientific, Waltham, MA, USA), in a humidified incubator at 37 °C and 5% CO_2_. Before tumour injections, cells were harvested with 0.05% trypsin–EDTA (25300-054, Thermo Fischer Scientific) and resuspended in D-PBS (14190-094, Thermo Fischer Scientific). 1 × 10^6^ MC38 cells in 100 µl were injected subcutaneously in the right flank of 6- to 7-week-old female C57BL/6J mice. Tumour volumes were evaluated with an electronic calliper (500-712-20, Mitutoyo, Kawasaki, Japan), and calculated with the formula *length x width*^2^ × *0.5*. Mice were sacrificed when tumour volume exceeded 1500 mm^3^, or when reporter expression was comparable to background. C57BL/6J mice were purchased from Charles River Laboratories (Saint Germain Nuelles, France) or bred at the KU Leuven Animal Research Center. All animal experiments were approved by the KU Leuven Animal Ethics Committee (P130/2017) and were performed in accordance with the regulations of the European Union and Belgium concerning the protection of laboratory animals.

### Intratumoral DNA transfection

Intratumoral electroporation was performed according to a previously described protocol^3,11^. 20 µg pDNA in 50 µl sterile milliQ water was injected intratumorally, immediately followed by two series of four 5-ms square-wave pulses of 600 V/cm in perpendicular directions at a frequency of 1 Hz. Electrical pulses were delivered by the NEPA21 Electroporator (Sonidel Limited, Dublin, Ireland) with CUY650P5 tweezer electrodes (Sonidel Limited) at a fixed width of 5 mm and covered with Eco Ultrasound Transmission Gel (G0066, Fiab, Vicchio, Italy). Pulse current and total energy were verified with the NEPA21 readout.

Complexes of pDNA with in vivo-jetPEI (201-10G, Polyplus-transfection, Illkirch, France) were prepared according to the manufacturer’s instructions. 20 µg pDNA and 3.2 µl in vivo-jetPEI (N/P = 8) were each diluted in 25 µl of 5% sterile D-glucose. Both dilutions were mixed, and following a 15-min incubation at room temperature, 50 µl was injected intratumorally per mouse.

For electroporation- and jetPEI-driven transfection, pDNA injection was performed manually with a syringe without thorough control of the injection speed. On average, injection of 50 µl lasted 10–20 s.

### Bioluminescence imaging

*F*luc expression was visualised and quantified by bioluminescence imaging (IVIS Spectrum, PerkinElmer, Waltham, MA, USA) at the Molecular Small Animal Imaging Center (MoSAIC) at KU Leuven. For in vivo imaging, mice were subcutaneously injected with 126 mg/kg D-luciferin substrate (E6551, Promega, Madison, WI, USA) at 15 mg/ml in D-PBS, after which bioluminescence intensity was measured every two minutes. Intratumoral *f*luc expression was defined as the maximal total radiance (in photons per second) measured in a specified region of interest covering the tumour area. Mice were anesthetised by isoflurane inhalation during the whole procedure. For ex vivo imaging, mice received a second subcutaneous injection with D-luciferin after in vivo bioluminescence imaging. Five minutes later, mice were sacrificed. Different organs were excised and analysed. *F*luc expression in the individual organs was calculated as the total bioluminescent signal measured in a region of interest covering the organ.

### Statistics

At the start of experiments, mice were randomised based on tumour volume and weight. Data of different groups were compared at different time points with Mann–Whitney tests. Outliers were detected with the Grubb’s test. Statistical analyses were performed with Graphpad Prism 8.4.3 (Graphpad Software, San Diego, CA, USA), with two-sided P values below 0.05 considered as significant.

## Data Availability

The datasets generated or analysed during the current study are available from the corresponding authors on reasonable request.
